# Duration of intervals in the care-seeking pathway of lung cancer in Nepal

**DOI:** 10.3332/ecancer.2025.1825

**Published:** 2025-01-16

**Authors:** Shama Pandey, Bishnu Dutta Paudel, Bibek Acharya, Sandhya Chapagain Acharya, Ambuj Karn, Saugat Poudyal, Manish Poudel, Pradeep Thapa, Jasmine Gurung, Ramila Shilpakar

**Affiliations:** 1 National Academy of Medical Sciences, Bir Hospital, Kathmandu 44600, Nepal; 2 KIST Medical College and Teaching Hospital, Lalitpur 44700, Nepal; 3 Tilganga Institute of Ophthalmology, Kathmandu 44600, Nepal

**Keywords:** delayed diagnosis, lung cancer, time factors

## Abstract

**Introduction:**

Patients with lung cancer in Nepal often present at an advanced stage. The purpose of this study was to determine the access, diagnostic and treatment intervals in patients with lung cancer and to identify factors that may be causing the delays leading to advanced presentation.

**Methods:**

This was a descriptive, cross-sectional study conducted in the Department of Clinical Oncology, Bir Hospital from July 2023 to April 2024 after obtaining ethical approval from the Institutional Review Board. Patients with newly diagnosed lung cancer were interviewed and data was collected. Data were presented in the forms of percentages and mean/median. Univariate and multivariate logistic regression analysis was done to assess the association between various factors and different delays.

**Results:**

Of the 100 patients included, 56% were men and the mean age was 64.5 ± 10.8 years. 64% of the patients had stage IV disease. The median access, diagnostic and treatment interval were 44.5 days, 45.5 days and 26 days, respectively. Access, diagnostic and treatment delays were seen in 72%, 63% and 42% of the patients, respectively. Receiving empirical anti-tubercular treatment and visiting informal healthcare providers as their first healthcare contact was associated with diagnostic delay whereas smoking was associated with treatment delay.

**Conclusion:**

There is a significant delay in the care-seeking pathway of lung cancer in Nepal. Implementing corrective measures to address these could help improve the outcomes for these patients.

## Introduction

Lung cancer is the most commonly diagnosed malignancy and the leading cause of cancer-related deaths in Nepal [1]. The majority of the patients present at an advanced stage [2].

Timely diagnosis and treatment of lung cancer is crucial. As per the World Health Organisation (WHO), cancer early diagnosis has been categorised into three sequential steps – access to care, evaluation and treatment; each with a corresponding interval: access interval, diagnostic interval and treatment interval, respectively [3]. The symptom-to-diagnosis interval is the sum of the access interval (symptom onset to first healthcare visit) and diagnostic interval (first healthcare visit to diagnosis). This has provided a background to identify and address issues regarding delay in cancer care.

Various factors contribute to delays in these durations in Nepal, a few being patients’ failure to recognise suspicious symptoms of cancer, lower socioeconomic status, challenging geographical landscape and distance from healthcare facilities, lack of proper referral system, longer waiting time for investigations and socio-cultural factors [4]. There are very few studies done in Nepal to assess delays in lung cancer. One such study conducted a decade ago, revealed that only 3% of the patients with lung cancer underwent resection. Advanced disease was the primary cause of unresectability in most patients. However, this study primarily focused on the surgical aspects of unresectability [5].

This study aims to determine the intervals in the care-seeking pathway of lung cancer patients and to identify the factors associated with delays. This information can guide interventions to reduce these delays.

## Methods

A descriptive, cross-sectional questionnaire-based study was conducted in the Department of Clinical Oncology, Bir Hospital, Nepal, from July 2023 to May 2024. The Institutional Review Committee of the National Academy of Medical Sciences provided the ethical approval. (Reference Number: 35/2080/81)

Patients aged 18 years and above with newly diagnosed lung carcinoma were enrolled in the study after obtaining written informed consent. Patients with extrapulmonary primary malignancy with metastasis to the lung were excluded.

### Convenience sampling was used

A questionnaire was developed to be appropriate for the study objectives and the Nepalese context. It was pilot-tested with 20 patients to ensure the questions were understandable. Information was collected based on face-to-face interviews using the questionnaire with the patient/their family members in a private room at the time of treatment initiation. The interview was conducted by the primary investigator. Patient’s demographics, including age, sex, address, education, smoking status and comorbidities (any pre-existing chronic illnesses) were recorded. The Eastern Cooperative Oncology Group Performance Status (ECOG PS) was determined by the treating oncologist at the time of treatment initiation. The first symptom at onset and the symptom that triggered the healthcare visit were noted. Histological diagnosis and stage of cancer were also documented. The choice of first healthcare provider, along with the date of first contact, the date of diagnosis (i.e., date of dispatch of the histopathological reports) and the date of treatment (i.e., date of treatment initiation) was noted. As patients usually keep a hard copy of their medical records (discharges and investigations), the dates were crosschecked from there to minimise recall bias. In case a mismatch of information was noted, the dates as written in the medical record were taken as the accurate date.

The access interval (interval between symptom onset to first visit to a healthcare provider), diagnostic interval (first visit to a healthcare provider to date of histological diagnosis) and treatment interval (diagnosis to initiation of treatment) were calculated in days. According to the WHO, the duration from onset of symptoms to treatment initiation should be less than 90 days in order to reduce delays in care and to optimise the effectiveness of treatment [3]. Based on the study done by Yohannan *et al* [6] in India, an access interval of more than 30 days and a diagnostic interval of more than 35 days were considered as access delay and diagnostic delay, respectively. A treatment interval exceeding 30 days or more was considered a treatment delay, based on another study done in India, which found a median treatment interval of 20 days [7].

Data were entered in Microsoft Excel 2016 and analysis was done by Statistical Package for Social Sciences software version 20. Frequencies/percentages were calculated for categorical variables and measures of central tendency were calculated for quantitative variables. For the association of categorical variables, chi-square test was used; if the expected cell value was less than 5, Fisher Exact test was used. For the average comparison of numerical variables, an independent *t*-test was used and Welch's *t*-test was used when unequal variances between the groups were observed. Univariate and multivariate logistic regression analyses were done to assess the association between various factors and different delays of interest. The univariate analysis included age, gender, area of residence (rural versus urban), education level, comorbidities, ECOG PS, current/former smoker, symptom at onset, a symptom that triggered the first healthcare visit, the first point of healthcare contact, being treated for tuberculosis and histological diagnosis. The significant variables from the univariate analysis were included in the multivariate logistic regression model, with the dependent variable being access, diagnostic or treatment delay. A *p*-value of less than 0.05 was considered statistically significant.

## Results

Out of 100 patients included in the study, the mean age was 64.5 ± 10.8 years (with a minimum of 27 years and a maximum of 88 years). Eighty-four (84%) of the patients were smokers with median pack years of 23 (IQR: 30–60). Patient demographics are summarised in Table 1.

The most common symptoms at onset and symptoms leading to the first healthcare visit are shown in Table 2.

None of the patients visited an oncologist as their first point of healthcare contact, 78% visited a trained medical professional, whereas the remaining 22% visited an informal healthcare provider (pharmacy/traditional healer).

**Figure 1.**
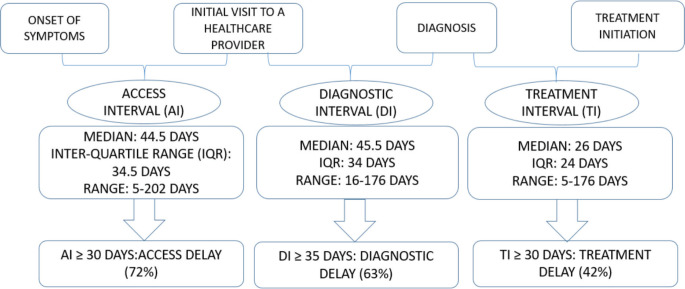
Access interval, diagnostic interval and treatment interval.

The longest interval was the diagnostic interval (median 45.5 days) followed by the access interval (median 44.5 days) as shown in Figure 1. Access delay, diagnostic delay and treatment delay were seen in 72%, 63% and 42% of the patients, respectively.

Univariate analysis showed a statistically significant association of access delay of 30 days or more with living in a rural area (*p* = 0.004), having low literacy or limited education (primary level) (*p* = 0.026) and having no comorbidities (*p* = 0.022) (Table 3). Multivariate analysis, however, did not show any significant association between access delay and the above-mentioned factors. (Table 4)

Being treated for tuberculosis (*p* = 0.002), living in a rural area (*p* = 0.017), having an informal healthcare provider as their first point of contact (*p* = 0.002) and age (*p* = 0.029) were shown to have a statistically significant association with diagnostic delay (≥35 days) in univariate analysis (Table 3). As ‘being treated for tuberculosis’ showed high multi-collinearity, it was excluded from the multiple logistic regression analysis. A significant association between having an informal healthcare provider as their first point of contact and diagnostic delay was confirmed by multivariate analysis (*p* = 0.022) (Table 4).

Furthermore, univariate analysis showed a statistically significant association of treatment delay (≥35 days) with living in rural areas (*p* = 0.018), having low literacy or limited education (*p* = 0.038) and being a current/former smoker (*p* = 0.04) (Table 3). A significant association between smoking and treatment delay was confirmed by multivariate analysis (*p* = 0.037) (Table 4).

Upon inquiry with the patients, the common reasons for access delay include misinterpretation of symptoms (36.4%) and financial difficulties (25%). Being treated for other diseases (30.7%) and long waiting times for investigations (17%) were the reasons for the diagnostic delay. Reasons for treatment delay include receiving alternative medicine (20.5%) and fear of adverse effects of cancer treatment (14.8%).

## Discussion

Lung cancer is the most common cancer in Nepal and places a significant healthcare burden. Delays in presentation, diagnosis and treatment of patients with lung cancer further complicate these challenges. In our study, the median access, diagnostic and treatment intervals were 44.5 days, 45.5 days and 26 days, respectively. The majority of patients (72%) had access delay, followed by diagnostic delay (63%) and treatment delay (42%). Being treated for tuberculosis and having an informal healthcare provider as their first point of healthcare contact was associated with diagnostic delay, whereas being a current/former smoker was associated with treatment delay.

In our study, the majority of the patients (84%) were smokers, which is consistent with studies from India, conducted by Yohannan *et al* [6] and Vashistha *et al* [7], where 84% and 77% of patients were current/former smokers, respectively. There is an urgent need to ramp up smoking cessation campaigns; the lack of smoking cessation clinics, drugs and quit helplines highlights the critical need for stronger programs. Prioritising tobacco control and smoking cessation is essential to reduce the burden of lung cancer [8]. The majority of patients had stage IV disease (64%) in our study, which is consistent with studies done in India(64%) [7], Brazil (65.2%) [9] and Singapore (67.8%) [10].

The symptoms of lung cancer may be due to the primary tumour, metastases or due to manifestations of paraneoplastic syndromes. In our study, common symptoms at onset were cough and chest pain; however, the common symptoms that triggered presentation to healthcare were difficulty in breathing and haemoptysis. Chronic smokers often attribute their cough to smoking, pollution or an acute respiratory infection. This can lead to a lower tendency to visit healthcare for the symptom, typically only visiting when another symptom arises and becomes a cause of concern. A similar pattern of false attribution of cough has also been shown in an Indian study [11].

The median access interval is 44.5 days (range 5–202 days), which is longer than in Bangladesh (median 10 days) [12], but similar to Ducze, Turkey (median 45 days) [13]. Access delay was seen in 72% of the patients, longer than in another study done in Istanbul, Turkey (48.5%) [14]. On multivariate analysis, none of the factors was associated with an access delay.

The median diagnostic interval is 45.5 days (range 16–176 days), which is shorter than in Bangladesh (107 days) [12], but longer than in Ducze, Turkey (10 days) [13]. Diagnostic delay was seen in 63% of the patients and 14% of the patients were empirically treated with anti-tubercular treatment. Being treated for tuberculosis led to diagnostic delay. A previous Nepalese study found that 17% of patients were receiving empirical anti-tubercular treatment which contributed to lung cancer management delays [15]. A similar finding was also reported in a study conducted in India [16]. Patients are often prescribed empirical anti-tubercular treatment and continue taking it for months, despite persisting symptoms, hoping for improvement. Furthermore, visiting an informal healthcare provider as their first point of contact was associated with diagnostic delay. Similar results were seen in Bangladesh where the intervals between symptom onset and diagnosis and treatment were significantly longer for patients who visited informal healthcare providers [12].

The median treatment interval is 26 days (range 5–176 days), which is longer than in Turkey (median 12 days) [13], but similar to that observed in India (median 21 days) [7]. Treatment delay was seen in 42% of our patients. Smoking was significantly associated with treatment delay in the multivariate analysis. A study done by Heiden *et al* [17] supported the link between smoking and treatment delay and found that compared to former smokers, current smokers had a longer treatment delay.

While numerous studies have investigated lung cancer delays worldwide, this study provides unique insights into the specific context of delays in Nepal. Our findings reveal significant delays in all three intervals particularly in the access and the diagnostic intervals. In LMICs like Nepal, patient factors, geographical factors and limited healthcare resources contribute to delays in accessing healthcare services and diagnosis. However, once diagnosed, more than half of the patients received treatment within a month. This highlights the importance of focusing interventions on addressing the challenges before diagnosis. There might be a large number of people with lung cancer having respiratory difficulties in rural areas who are undiagnosed and being treated by informal healthcare providers. Strengthening primary healthcare services and training community healthcare workers can help to identify these patients. People who are current or former smokers and those diagnosed with Chronic Obstructive Pulmonary Diseases (COPD) should be educated about the warning signs of lung cancer and encouraged to visit a formal healthcare provider if such symptoms arise, instead of visiting an informal one. Healthcare workers working in non-cancer centers should be trained to recognise warning signs. As computed tomography scans are not widely available in Nepal, all patients with suspected symptoms should at least receive a chest X-ray. Careful consideration needs to be given to patients receiving anti-tubercular treatment. If symptoms persist or worsen after a month of treatment, further investigation is warranted to rule out malignancy or a referral to a higher level healthcare facility. Furthermore, this study provides evidence to advocate for policy-level interventions that strengthen cancer-related healthcare services and manpower.

We acknowledge that there are a few limitations to this study. First, it is dependent on the patients’/family members’ history regarding the date of onset of symptoms, and recall bias must be accounted for while interpreting the results. However, these data appear consistent with that seen in our daily clinical practice. Furthermore, the sample size is relatively small and we have enrolled only those patients who visit our center, there may be many more in the care-seeking pathway who might never visit an oncologist or a tertiary center for further diagnosis/treatment. Additionally, selection bias is a potential concern, as bedridden patients, who might have longer delays might be unable to visit our center. As it is a single-center study, the results might not be generalised to the entire population.

## Conclusion

There is a significant delay in the care-seeking pathway of lung cancer, mostly in the access and the diagnostic interval. Chronic smokers and patients with COPD should be warned about the alarming signs of lung cancer. For healthcare workers, it is of utmost importance to rule out lung cancer in patients with high suspicion. Patients clinically diagnosed with tuberculosis and under empirical anti-tubercular treatment should be vigilantly monitored. Finally, there is a need for targeted interventions to address these barriers.

## Conflicts of interest

None.

## Funding

None.

## Figures and Tables

**Table 1.** Patient demographics (*n* = 100).

	*n* (%)
Sex Male Female	56 (56.00%) 44 (44.00%)
Address Urban Rural	52 (52.00%) 48 (48.00%)
Education Illiterate & Primary School Secondary School and above	57 (57.00%) 43 (43.00%)
Comorbidities Diabetes Hypertension Heart diseases COPD Others	6 (10.00%) 15 (15.00%) 5 (5.00%) 21 (21.00%) 3 (3.00%)
ECOG PS 0 1 2 3	16 (16.00%) 36 (36.00%) 39 (39.00%) 9 (9.00%)
Histological diagnosis Adenocarcinoma Squamous cell carcinoma Small cell carcinoma Others	37 (37.00%) 47 (47.00%) 15 (15.00%) 1 (1.00%)
Stage at diagnosis Stage I Stage III Stage IV	4 (4.00%) 32 (32.00%) 64 (64.00%)

**Table 2.** Symptoms at onset and symptoms that led to presentation to healthcare (*n* = 100).

Symptom	At onset *n* (%)	At presentation *n* (%)
Cough	50 (50.00)	16 (16.00)
Difficulty in breathing	13 (13.00)	26 (26.00)
Chest pain	16 (16.00)	17 (17.00)
Hemoptysis	7 (7.00)	25 (25.00)
Hoarseness of voice	3 (3.00)	2 (2.00)
Fever	1 (1.00)	4 (4.00)
Others	10 (10.00)	10 (10.00)
Total	100 (100.00)	100 (100.00)

**Table 3.** Univariate analyses of various factors with access, diagnostic and treatment delay.

Variables	Category	Access delay		Diagnostic delay		Treatment Delay	
		Number (%)	*p* value	Number (%)	*p* value	Number (%)	*p* value
Sex	Male	40 (55.6)	0.886	33 (52.4)	0.341	21 (50)	0.304
	Female	32 (44.4)		30 (47.6)		21 (50)	
	Total	72 (100)		63 (100)		42 (100)	
Urban/Rural	Urban	31 (43.1)	0.004	27 (42.9)	0.017	16 (38.1)	0.018
	Rural	41 (56.9)		36 (57.1)		26 (61.9)	
	Total	72 (100)		63 (100)		42 (100)	
Education	Secondary and higher	26 (36.1)	0.026	23 (36.5)	0.087	13 (31)	0.038
	Illiterate/Primary	46 (63.9)		40 (63.5)		29 (69)	
	Total	72 (100)		63 (100)		42 (100)	
Comorbidities	Yes	21 (29.2)	0.022	19 (30.2)	0.112	14 (33.3)	0.636
	No	51 (70.8)		44 (69.8)		28 (66.7)	
	Total	72 (100)		63 (100)		42 (100)	
Histological diagnosis	Adenocarcinoma	25 (34.7)	0.727	26 (41.3)	0.204	16 (38.1)	0.406
	Squamous cell carcinoma	36 (50)		30 (47.6)		17 (40.5)	
	Small cell carcinoma	10 (13.9)		7 (11.1)		8 (19)	
	Others	1 (1.4)		0 (0)		1 (2.4)	
	Total	72 (100)		63 (100)		42 (100)	
Symptom at onset	Cough	39 (54.2)	0.516	32 (50.8)	0.721	22 (52.4)	0.37
	Difficulty in breathing	10 (13.9)		7 (11.1)		5 (11.9)	
	Chest Pain	11 (15.3)		9 (14.3)		5 (11.9)	
	Hemoptysis	4 (5.6)		5 (7.9)		2 (4.8)	
	Hoarseness of voice	2 (2.8)		1 (1.6)		3 (7.1)	
	Fever	1 (1.4)		1 (1.6)		1 (2.4)	
	Others	5 (6.9)		8 (12.7)		4 (9.5)	
	Total	72 (100)		63 (100)		42 (100)	
Symptom at presentation	Cough	10 (13.9)	0.298	8 (12.7)	0.332	7 (16.7)	0.537
	Difficulty in breathing	22 (30.6)		19 (30.2)		13 (31)	
	Chest Pain	12 (16.7)		8 (12.7)		6 (14.3)	
	Hemoptysis	17 (23.6)		16 (25.4)		8 (19)	
	Hoarseness of voice	2 (2.8)		1 (1.6)		2 (4.8)	
	Fever	4 (5.6)		4 (6.3)		1 (2.4)	
	Others	5 (6.9)		7 (11.1)		5 (11.9)	
	Total	72 (100)		63 (100)		42 (100)	
Treated for TB	Yes	10 (13.9)	1.00	14 (22.2)	0.002	3 (7.1)	0.093
	No	62 (86.1)		49 (77.8)		39 (92.9)	
	Total	72 (100)		63 (100)		42 (100)	
Smoker	Yes (Current/Former)	62 (86.1)	0.262	50 (79.4)	0.099	39 (92.9)	0.04
	No	10 (13.9)		13 (20.6)		3 (7.1)	
	Total	72 (100)		63 (100)		42 (100)	
First point of contact	Informal healthcare provider	18 (25)	0.246	20 (31.7)	0.002	11 (26.2)	0.389
	Trained medical professional	54 (75)		43 (68.3)		31 (73.8)	
	Total	72 (100)		63 (100)		42 (100)	
ECOG PS	0	8 (11.1)	0.198	9 (14.3)	0.812	7 (16.7)	0.560
	1	27 (37.5)		23 (36.5)		13 (31)	
	2	31 (43.1)		26 (41.3)		17 (40.5)	
	3	6 (8.3)		5 (7.9)		5 (11.9)	
	Total	72 (100)		63 (100)		42 (100)	
Age	Mean (SD)	64.4 (10.7) Versus 64.9 (11.2)	0.835	62.7 (11.4) Versus 67.6 (9)	0.029	66.1 (8.4) Versus 63.4 (12.2)	0.180

**Table 4.** Multiple logistic regression analysis.

Variables	Univariate logistic regression		Multiple logistic regression	
	OR (95% CI)	*p* value	OR (95% CI)	*p* value
**Access delay**				
Rural (compared with Urban)	3.97 (1.50 to 10.51)	0.004	2.73 (0.86 to 8.71)	0.089
Illiterate and primary education (compared with Secondary and Higher	2.73 (1.11 to 6.71)	0.026	1.61 (0.54 to 4.77)	0.395
No comorbidities (compared with comorbidities)	2.80 (1.14 to 6.89)	0.022	2.44 (0.95 to 6.28)	0.063
**Diagnostic delay**				
Rural (compared with Urban)	2.78 (1.19 to 6.50)	0.017	0.54 (0.22 to 1.3)	0.167
Age	0.954 (0.91 to 0.996)	0.029	1.01 (0.99 to 1.02)	0.178
Traditional healers and Pharmacy (compared with trained medical professionals)	8.14 (1.78 to 37.23)	0.002	8.99 (1.81 to 44.66)	**0.022**
**Treatment delay**				
Rural (compared with Urban)	2.66 (1.17 to 6.03)	0.018	2.49 (0.9 to 6.9)	0.079
Illiterate and primary education (compared with Secondary and Higher)	2.39 (1.04 to 5.50)	0.038	1.41 (0.5 to 3.94)	0.513
Smoker (compared with never-smoker)	3.76 (0.997 to 14.15)	0.04	4.32 (1.09 to 17.12)	**0.037**

A *p*-value of less than 0.05 was considered statistically significant.
